# Unveiling the Differentiation Potential of Ovarian Theca Interna Cells from Multipotent Stem Cell-like Cells

**DOI:** 10.3390/cells13151248

**Published:** 2024-07-25

**Authors:** Hanne Vlieghe, Maria João Sousa, Dania Charif, Christiani A. Amorim

**Affiliations:** Pôle de Recherche en Physiopathologie de la Reproduction, Institut de Recherche Expérimentale et Clinique, Université Catholique de Louvain, Avenue Hippocrate 54, bte B1.55.03, 1200 Brussels, Belgium; hanne.vlieghe@uclouvain.be (H.V.); maria.sousa@uclouvain.be (M.J.S.); dania.charif@student.uclouvain.be (D.C.)

**Keywords:** theca interna cells, multipotent mesenchymal stem cells, differentiation, ovarian follicle development, steroidogenesis

## Abstract

Research question: Theca interna cells (TICs) are an indispensable cell source for ovarian follicle development and steroidogenesis. Recent studies have identified theca stem cells (TSCs) in both humans and animals. Interestingly, TSCs express mesenchymal stem cell (MSC)-related markers and can differentiate into mesenchymal lineages. MSCs are promising for tissue engineering and regenerative medicine due to their self-renewal and differentiation abilities. Therefore, this study investigated the potential origin of TICs from MSCs. Design: Whole ovaries from postmenopausal organ donors were obtained, and their cortex was cryopreserved prior to the isolation of stromal cells. These isolated cells were differentiated in vitro to TICs using cell media enriched with various growth factors and hormones. Immunocytochemistry, an enzyme-linked immunosorbent assay, flow cytometry, and reverse transcription–quantitative polymerase chain were employed at different timepoints. Data were analyzed using one-way ANOVA. Results: Immunocytochemistry showed an increase in TIC markers from day 0 to day 8 and a significant rise in MSC-like markers on day 2. This corresponds with rising androstenedione levels from day 2 to day 13. Flow cytometry identified a decreasing MSC-like cell population from day 2 onwards. The CD13+ cell population and its gene expression increased significantly over time. NGFR and PDGFRA expression was induced on days 0 and 2, respectively, compared to day 13. Conclusions: This study offers insights into MSC-like cells as the potential origin of TICs. Differentiating TICs from these widely accessible MSCs holds potential significance for toxicity studies and investigating TIC-related disorders like polycystic ovary syndrome (PCOS).

## 1. Introduction

**Figure 1:**
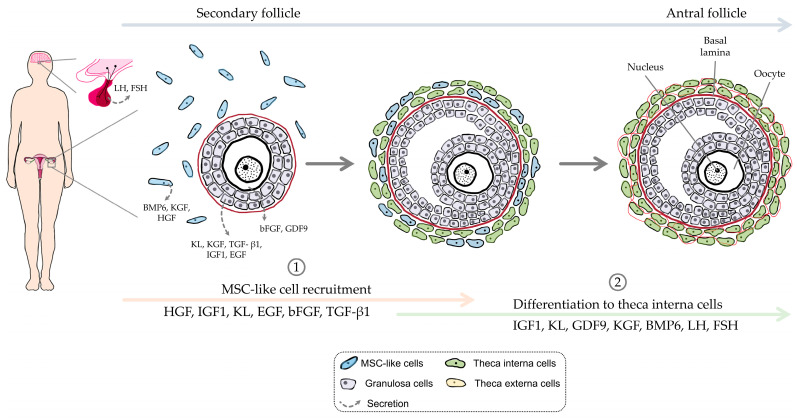
Schematic representation of TIC differentiation.

Theca cells, surrounding human follicles during the secondary stage of development and beyond, play a crucial role in providing structural support as well as facilitating steroidogenesis, which is essential for follicle survival and maturation [1]. As the theca layer is composed of both theca interna cells (TICs) and theca externa cells (TECs), it is specifically the internal layer that is responsible for the production of androstenedione, and thus, theca cells in this internal layer play a crucial role in steroidogenesis [2]. While their recruitment, differentiation (Figure 1), and function in steroidogenesis are well-established, ongoing research seeks to elucidate remaining uncertainties, particularly regarding the enigmatic cellular origin of TICs [3].

Ovarian progenitors or stem cells have already been pinpointed while investigating the origin of TICs. Research on stem cells in human ovaries indicated the presence of oogonial stem cells (OSCs), which are characterized by markers such as DDX4, VASA, and DAZL. Such OSCs have been isolated from both adult and postmenopausal ovaries and have demonstrated the capability to differentiate into oocyte-like cells [4,5,6,7,8]. Nevertheless, their role in TIC recruitment and differentiation has not been established. Instead, previous research suggests that a different progenitor or stem cell type may serve as the source of TICs. Our study focuses on these distinct progenitor cells and their relevance to the differentiation of TICs, thereby providing a more targeted investigation into their origins and potential applications.

In human ovaries, Dalman et al. [9] isolated a theca stem cell (TSC) population within the theca cell layer of small antral follicles. These specific TSCs have the capability to undergo differentiation into various mesenchymal cell lineages, including osteogenic, adipogenic, and chondrogenic lineages, as well as into theca progenitor cells in vitro. Moreover, they were characterized by the presence of CD29, CD44, CD73, CD90, and CD105 and the absence of CD45/34 [9]. The same expression pattern, together with a positive expression of POUF5F1, was observed in TSCs isolated from antral follicles of adult sheep ovaries [10]. These TSCs could also be differentiated into adipocyte- and osteocyte-like cells, as well as theca progenitor cells [10]. Also, TSCs isolated from porcine antral follicles exhibited similar characteristics to those isolated from sheep [11]. In cynomolgus monkeys, TSCs were obtained through the isolation and in vitro culture of ovarian cortex stromal cells, with characterization based on the presence of PDGFRA, CD271, and NESTIN [12]. These markers are known markers for Leydig stem cells, which are the male counterparts of TICs [13,14,15]. Recently, Wen et al. [16] showed the isolation and differentiation potential of both LY6A+ and PDGFRA+ cells found in the interstitial compartment of adult mice ovaries. This study showed proof of the differentiation capacity of ovarian MSC-like cells towards TICs. 

The above-mentioned makers that characterize TSCs portray this cell type as remarkably analogous to mesenchymal stem cells (MSCs). As described by the Mesenchymal and Tissue Stem Cell Committee of the International Society for Cellular Therapy, MSCs exhibit three minimal criteria. Firstly, they demonstrate plastic adherence during standard in vitro culture. Secondly, they express the surface markers CD73, CD90, and CD105 and lack CD45, CD34, CD14 or CD11b, CD79α or CD19, and HLA-DR. Finally, they can undergo differentiation into adipocytes, osteoblasts, and chondrocytes in vitro [17]. In addition, PDGFRA, CD271, and NESTIN are also known markers of MSCs [18,19,20]. Due to their ability to self-renew and differentiate, multipotent MSCs are a promising cell source for regenerative medicine and tissue engineering [21]. Moreover, recent research has revealed their potential to differentiate into neural lineages [22]. For over 60 years, researchers have effectively isolated MSCs from a wide range of sources, encompassing adult tissues, such as bone marrow, dental pulp, adipose tissue, and liver tissue, as well as perinatal tissues like the placenta, umbilical cord, and amniotic fluid [23,24,25,26,27]. Notably, adipose-derived MSCs (ADSCs) are particularly accessible as adipose tissue can be obtained through minimally invasive procedures, such as liposuction or surgical resection. 

Building upon the abovementioned resemblances between TSCs and MSCs, we hypothesized that TSCs represent a distinct subset of MSCs equipped with the ability to differentiate into TICs upon exposure to specific TIC-related growth factors and hormones. The goal of our study is to investigate the presence of MSCs during the in vitro differentiation process of TICs from postmenopausal ovarian cells and evaluate their potential role as the source of TICs. Identifying the origin of TICs and their formation of MSC-like cells within the ovarian interstitial component is crucial, as it can advance in vitro studies on TICs as well as our understanding of TIC-related disorders. 

## 2. Materials and Methods

### 2.1. Isolation of Ovarian Stromal Cells 

Whole ovaries were obtained from three postmenopausal multiorgan donors aged 50 to 64 years. The use of human ovarian tissue was approved by the University’s ethics board on 25 May 2019 (IRB reference 2018/19DEC/475). The ovaries were transported under controlled temperature conditions in Dulbecco’s phosphate-buffered saline (DPBS; Thermo Fisher Scientific, Merelbeke, Belgium). Upon arrival at our laboratory, the ovaries were first cut in half, after which the medullary component was removed using a scalpel blade and scissors. Subsequently, the remaining cortex was sectioned into fragments and cryopreserved, as described by Lierman et al. [28]. Following the controlled thawing of the cryopreserved ovarian samples, stromal cells were isolated [29]. Briefly, the tissue was first mechanically minced using a tissue chopper (Mickel Laboratory, Guildford, UK) set to a thickness of 0.5 mm and then submitted to an enzymatic digestion step using 0.28 Wünsch units/mL Liberase DH (Sigma-Aldrich, Darmstadt, Germany) and 8 Kunitz units/mL DNAse I (Sigma-Aldrich) at a temperature of 37 °C for 75 min with gentle agitation and pipetting every 15 min. Next, this enzymatic reaction was inactivated by adding DPBS supplemented with 10% heat-inactivated fetal bovine serum (FBS; Thermo Fisher Scientific). Finally, the obtained cell suspension was centrifuged at 350 g for 10 min, subsequent to its passage through 80 nylon net filters (Millipore, Overijse, Belgium) [30]. Cells were counted and divided for in vitro differentiation to TICs, RT-qPCR, flow cytometry, and immunocytochemistry. 

### 2.2. Differentiation of TICs

To induce the differentiation of the isolated ovarian stromal cells into TICs, the cells were initially seeded at a density of 80,000 cells per 1.9 cm^2^ on a collagen-coated plate. The culture medium consisted of Dulbecco’s modified Eagle’s medium/Ham’s nutrient mixture F-12 + GlutaMAX (DMEM/F12; Thermo Fisher Scientific) supplemented with 10% KnockOut GibcoTM serum replacement (KSR; Thermo Fisher Scientific), 1% antibiotic–antimycotic solution (Anti–Anti; Thermo Fisher Scientific), and additional growth factors and hormones (Table 1), as described by Asiabi et al. [31]. The cells were in vitro cultured at 37 °C in a humidified incubator with 5% CO_2_, and 50% of the medium was changed every 2 days. Throughout the culture period, samples of the cells and their media were collected at the following specific intervals: day 0 (before in vitro culture), day 2, day 8, and day 13. At each different timepoint, the cells were detached using Accutase^TM^ (Sigma-Aldrich), and their media were stored at −80 °C for subsequent analyses. 

### 2.3. Enzyme-Linked Immunosorbent Assay (ELISA)

Frozen-thawed culture media were utilized to quantify the levels of dehydroepiandrosterone (DHEA), progesterone (Enzo Life Sciences BVBA, Farmingdale, NY, USA), and androstenedione (ASD) (Bio-Connect, Huissen, The Netherlands) using an enzyme-linked immunosorbent assay according to the manufacturer’s instructions. Measurements were carried out in duplicates, and both blank and negative controls were included. 

### 2.4. RT-qPCR Analysis

To assess the presence of TICs and MSCs during the differentiation of ovarian cells to TICs, a reverse transcription–quantitative polymerase chain reaction (RT-qPCR analysis) was performed for specific TIC and MSC markers. For this purpose, detached cells were washed twice with DPBS and snap-frozen as a cell pellet, which was stored at −80 °C until RT-qPCR analysis. Upon usage, the snap-frozen cells were assessed for their relative mRNA levels. Total RNA extraction was performed using a GeneJet RNA Purification Kit (Invitrogen, Thermo Fisher Scientific), and reverse transcription into complementary DNA was accomplished using qScript™ cDNA SuperMix (Quanta Biosciences, Gaithersburg, MA, USA). A quantitative real-time polymerase chain reaction was carried out with TaqMan probes (Applied Biosystems, Waltham, MA, USA) (Table 2) and the TaqMan Gene Expression Master Mix (Applied Biosystems) on a StepOne Real-Time PCR System (Applied Biosystems). The housekeeping genes were selected based on previous RT-qPCR experiments in our laboratory [32]. The relative gene expression of the target genes was calculated using the Pfaffl method [33]. 

### 2.5. Flow Cytometry 

Detached cells were washed with DPBS and incubated for 10 min at room temperature with a set of well-known MSC markers [17], as well as CD13, which is a known marker for TICs [34] (Table 3). Next, the cells were analyzed by the BD FACSCanto™ II Clinical Flow Cytometry System (BD Biosciences, Erembodegem, Belgium). Further analysis was carried out using FlowJo software v10.10 (BD Biosciences).

### 2.6. Immunocytochemistry

Detached cells were transferred to a round coverslip coated with poly-L-lysine (Sigma-Aldrich). They were subsequently washed 3 times for 5 min with DPBS and fixed in 4% paraformaldehyde (Sigma-Aldrich) for 15 min at room temperature. Following fixation, the cells were permeabilized with 0.1% Triton X-100 (Sigma-Aldrich) for 10 min and blocked in 5% normal goat serum (NGS) for 1 h at room temperature. Then, they were incubated with the primary antibodies (Table 4) overnight at 4 °C. On the following day, Alexa-Fluor-conjugated secondary antibodies (Thermo Fisher Scientific) were added at a 1:200 dilution for 1 h at room temperature in the dark. In a final step, Hoechst 33342 (1:2000; Thermo Fisher Scientific) was added for 15 min at room temperature. Confocal imaging and image analysis were performed using a laser scanning microscope (LSM) 800 (Zeiss, Jena, Germany) and the ZenBlue Software v3.10 (Zeiss).

### 2.7. Statistical Analysis 

All statistics were carried out using GraphPad Prism 8 (GraphPad Software Inc., Solana Beach, CA, USA). The data were subjected to one-way ANOVA analyses, and statistical difference were determined through *p*-values below 0.05. Quantitative data are presented as mean ± SD, represented by error bars on the graphs. Three replicated experiments were used for analysis, and outliers were excluded from the datasets. 

## 3. Results

### 3.1. Hormone Secretion

**Figure 2:**
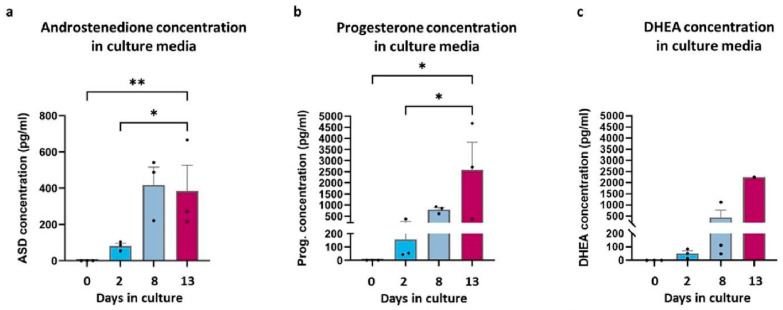
ELISA assay results. The concentration of androstenedione (**a**), progesterone (**b**), and DHEA (**c**) in pg/mL at different culture timepoints (* *p* < 0.05, ** *p* < 0.01).

The concentration of androstenedione during in vitro culture showed a notable increase towards the end of the culture, with levels rising from day 2 to day 13 (81.31 ± 25.09 pg/mL vs. 384.29 ± 245.82 pg/mL) (Figure 2a). This increase aligns with a significant augmentation in progesterone levels (155.79 ± 186.02 pg/mL (day 2) vs. 2582.77 ± 2154.83 pg/mL (day 13)) and a concurrent trend in DHEA concentrations from day 2 to day 13 (51.17 ± 35.23 pg/mL vs. 2244.34 pg/mL) (Figure 2b,c). 

### 3.2. RT-qPCR

**Figure 3:**
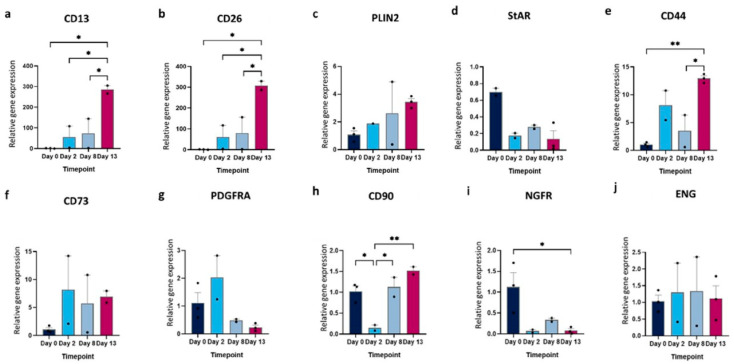
RT-qPCR analysis illustrating the relative expression of specific TIC- and MSC-related genes. Genes CD13, CD26, PLIN2, and StAR were chosen for TIC-related cell types (**a**–**d**), while CD44, CD73, PDGFRA, CD90, NGFR, and ENG were selected for MSC-like cells during in vitro differentiation (**e**–**j**) (* *p* < 0.05, ** *p* < 0.01).

To monitor changes in gene expression during the differentiation, CD13, CD26, PLIN2, and StAR were selected to detect shifts in cell type over time, specifically for TICs and luteal cells. The relative gene expression of the TIC-specific marker CD13 was significantly induced over time (1.38 ± 0.97 vs. 285.80 ± 27.43 on days 0 and 13, respectively) (Figure 3a). In line with the rise in CD13, an induction of CD26 was observed. The expression rose significantly from 1.35 ± 0.94 to 307.8 ± 29.54 at the start and the end of the differentiation, respectively (Figure 3b). The gene expression of PLIN2 appeared to follow this increase over time, although the increase was less pronounced than that of CD13 and CD26, and no significant differences were observed between days 0 and 13 (1.08 ± 0.49 vs. 3.44 ± 0.43) (Figure 3c). As opposed to the previous genes, the expression of StAR was repressed toward the end of the differentiation, decreasing significantly from 1.15 ± 0.77 at the beginning of the culture to 0.18 ± 0.03 on day 2 and further reducing to 0.14 ± 0.17 by the end of the in vitro culture (Figure 3d). 

For mesenchymal stem cells, CD44, CD73, CD90, ENG, PDGFRA, and NGFR (CD271) were chosen for the detection of MSC-like cells. CD44, expressed on MSCs, as well as on other cell types like fibroblasts and smooth muscle cells, showed a slight insignificant increase from day 0 to day 2 (1.05 ± 0.40 vs. 8.12 ± 3.75). Overall, the expression of this maker was induced from the beginning to the end of the culture (1.05 ± 0.40 vs. 12.94 ± 0.80) (Figure 3e). A similar, although again insignificant, increase was observed in the expression of CD73 from day 0 to day 2 (1.09 ± 0.59 vs. 8.18 ± 8.57). However, the expression level of this marker remained stable during the last days of the culture (Figure 3f). Although there was an increase in PDGFRA expression on day 2 (2.03 ± 1.11), this rise showed no significant difference compared to days 0, 8, or 13 (1.11 ± 0.63; 0.49 ± 0.07; and 0.24 ± 0.14, respectively). In contrast, the expression of CD90 and NGFR significantly decreased on day 2 (0.15 ± 0.10 for CD90 and 0.07 ± 0.05 for NGFR), compared to day 0 (1.02 ± 0.24 for CD90 and 1.12 ± 0.60 for NGFR). While the expression of CD90 increased again towards the end of the culture (0.52 ± 0.13 on day 13), the expression of NGFR remained low until day 13 (0.08 ± 0.08) (Figure 3g,h). The expression of ENG, present in many stromal cell types, did not show any significant difference over time (Figure 3j). 

### 3.3. Flow Cytometry 

**Figure 4:**
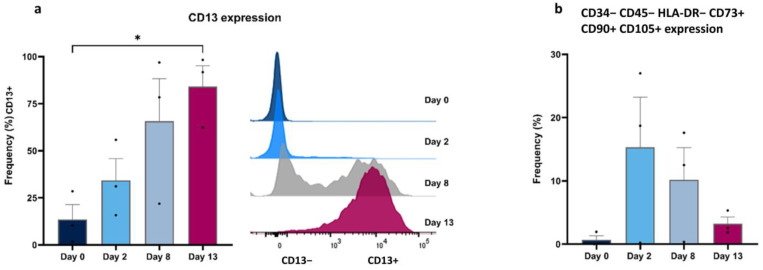
Flow cytometry analysis of specific TIC- and MSC-related membrane markers. (**a**) TICs were analyzed by the presence of CD13, graphically represented as the mean of CD13+ cells (left), with peaks of CD13− and CD13+ cells shown for one representative replicate (right). (**b**) MSC-like cells were analyzed by the absence of CD34, CD45, and HLA-DR and the presence of CD90, CD73, and CD105 (* *p* < 0.05).

Different analyses were performed using different cell marker combinations in FlowJo Software. For TIC analysis, the expression of CD13 was assessed over time. Starting from a negligible fraction at day 0 (13.52 ± 7.9%), the expression level of CD13 exhibited a significant escalation to 65.73 ± 22.56% by day 6 and increased significantly by day 13 (84.13 ± 11.08) (Figure 4a). In the analysis of MSC-like cells, cells lacking CD34, CD45, HLA-DR, and expressing CD73, CD90, and CD105 were selected. This population of cells represented less than 1% on day 0 but increased to 15.30 ± 7.9% on day 2, after which it decreased towards the end of the culture (3.2 ± 1.1% on day 13). This observed trend suggests the presence of an MSC-like cell population, specifically noticeable on day 2 (Figure 4b). 

### 3.4. Immunocytochemistry

**Figure 5:**
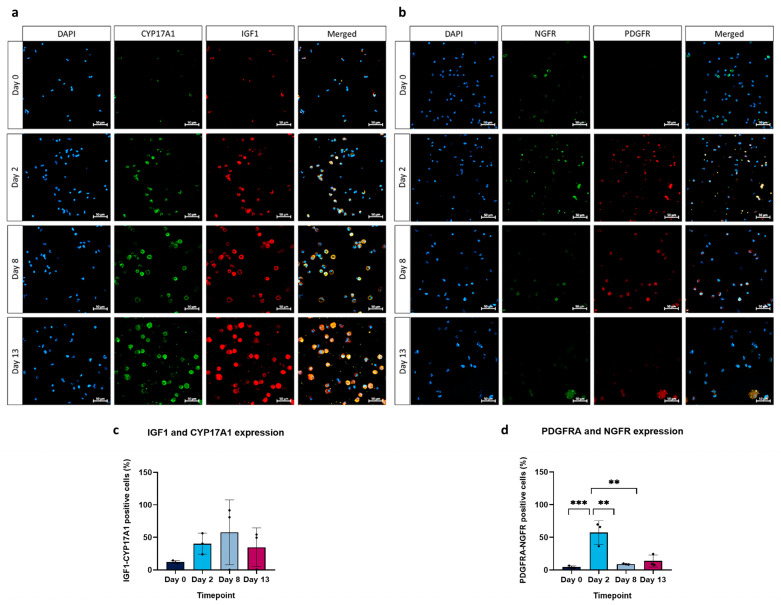
Immunofluorescence staining results. Representative images illustrating the proportion of cells positive for CYP17A1 and IGF1 (**a**) as well as for NGFR and PDGFRA (**b**). Double-positive cells from all replicates were counted from three representative images and divided by the total number of cells. This is graphically represented for CYP17A1 and IGF1 (**c**) and for NGFR and PDGFRA (**d**) (** *p* < 0.01, *** *p* < 0.001).

Makers specific to TICs were combined in one staining, while those indicative of MSC-like cells were combined in a separate staining. Three representative images were taken, and the quantification of double-positive cells was counted. The results unveiled an increase in the proportion of cells positive for theca cell markers from day 0 to day 8 (12.13 ± 2.11% vs. 57.73 ± 49.99%) (Figure 5a), while the proportion of MSC-like cell markers increased significantly from day 0 to day 2 (4.54 ± 1.71% vs. 57.34 ± 18.05%), and showed a decrease from day 0 towards the end of the culture (57.34 ± 18.05% vs. 8.86 ± 0.94% and 13.64 ± 9.22%, day 8 and 13, respectively) (Figure 5b). 

## 4. Discussion

This study aimed to investigate the potential of MSC-like cells as precursors of TICs, as well as to analyze TICs’ differentiation from stromal cells at different timepoints of in vitro culture. This builds upon existing research aimed at identifying and isolating progenitor or stem cells of TICs in the ovary [9,13,16,31,35,36,37], with a specific focus on ovarian stromal cells’ differentiation towards TICs and the exploration of MSC-like cells as a potential source of theca stem cells. As the differentiation of ovarian MSC-like cells into steroidogenic theca cells was recently shown by Wen et al. [16] in mice, our study shows the presence of ovarian MSC-like cells and their differentiation towards TICs in humans. 

Employing criteria defined by Dominici et al. [17], we applied multiple methods to investigate genes and proteins expressed in MSC-like cells. Flow cytometry analyses using selected markers for MSC-like cells unveiled a significant increase in MSC-like cells at day 2, declining toward the end of the culture. Interestingly, this cell population was not detected on day 0 among the ovarian cells soon after isolation. While, in principle, this could challenge our hypothesis, it is crucial to consider that cells typically require at least 24 h in culture to express all their surface markers after a process of freezing and thawing [38]. Nevertheless, a population of MSC-like cells emerged after 2 days and decreased as the proportion of CD13-positive TICs increased. This strong increase was also evident in the immunohistochemistry results, where the proportion of PDGFRA and NGFR double-positive cells was the highest after 2 days of in vitro culture. Both PDGFRA and NGFR were found in thecal stem cells isolated from non-human primates [12]. In humans, these two markers are known to be expressed in Leydig stem cells [14,15] found in testicular tissue, which gives rise to Leydig cells, the male counterpart of TICs [39]. The observed gene expression of PDGFRA mirrored an increase in expression on day 2, but this was not the case for NGFR, which was significantly higher only on day 0. Several processes influencing transcription and translation, coupled with protein stability, contributed to the inconsistency between relative gene expression and protein levels–varying up to 40%, depending on the tissue and in vitro culture method [40]. Furthermore, CD44 gene expression showed an elevation on both days 2 and 13. CD44 is known to be expressed in granulosa and luteal cells [41], but its presence on TICs remains unknown as an in-depth characterization of these cells has not been carried out yet. Gene expressions of fibroblast and MSC-like cell markers CD73 and CD90 exhibited inconsistent patterns. Nevertheless, as these markers are expressed on several cell types, drawing conclusions based on a single gene’s expression is challenging. Also, the expression of these markers was not specifically investigated in TICs. Based on our findings, theca cells might express CD90 and CD73. The gene expression of ENG remained similar throughout the culture period and was not specific enough to draw conclusions. While some research groups indicated the absence of MSC-like cells in the ovary, we hypothesized that these cells are present in the ovary and are potential progenitors of TICs. Indeed, we identified such a group of cells after two days of culture. As indicated by Dadashzadeh et al. [42], the type of cell culture media supplementation can influence the selection, proliferation, and differentiation of cells over time. Therefore, we suspect that the small population of MSC-like cells, detected on day 0, is selected and proliferates in the first few days of culture, leading to their subsequent differentiation towards TICs. This selection phenomenon was also observed after isolating and culturing ovarian stromal cells from adult women [43]. This proposed hypothesis can be confirmed by future in vivo studies in mice.

Furthermore, looking into the presence of TICs during in vitro differentiation, we noted their absence within stromal cells at the initiation of the culture (day 0). This observation is consistent with the understanding that theca cells emerge as a distinct layer surrounding follicles starting from the secondary stage onward, which is a feature no longer present in postmenopausal ovaries [44]. Despite previous research showing that TICs typically differentiate from postmenopausal ovarian stromal cells within an 8-day timeframe, their presence was unexpectedly detected as early as two days of in vitro culture [31]. This appearance coincided with a subtle increase in hormone levels and an augmented percentage of cells expressing both TIC markers, CYP17A1 and IGF1. Moreover, the gene and protein expressions of CD13 were consistent with these findings. The differentiation of TICs at earlier than 8 days of culture was also shown in TICs that differentiated from bovine ovarian stromal cells, with TICs detectable as early as 48 h into in vitro culture [45]. Interestingly, cells cultured beyond day 8 showed signs of further maturation and differentiation towards small luteal cells. These cells displayed both gene and protein expressions of the TIC marker CD13, along with the induced gene expression of both CD26 and PLIN2, which are recognized markers in luteal cells [46,47]. However, the identification of the luteal marker StAR diverged from these observations as its level remained relatively constant throughout the culture period. This discrepancy can be attributed to StAR’s expression not only in TICs but also in luteal cells present in the postmenopausal ovary [48]. 

In conclusion, these findings represent an initial step toward unraveling the intricate dynamics of TIC differentiation, potentially derived from MSC-like cells. 

Differentiating TICs from MSC-like cells holds significant potential for both toxicity studies and therapeutic applications, such as treating TIC-related disorders like polycystic ovary syndrome (PCOS). However, the observed differences in expression patterns between TICs and MSCs suggest that the differentiated TICs may not fully recapitulate native TICs’ functionality. It is essential to assess whether the MSC-derived TICs can support folliculogenesis as effectively as native TICs. Comparative studies at the transcriptomic and proteomic levels between native TICs and MSC-derived TICs are necessary to identify critical differences and refine differentiation protocols. This will enhance our understanding of the molecular mechanisms underlying TIC differentiation and function.

Additionally, implementing in vivo models to validate the functionality of MSC-derived TICs is crucial. Such studies can demonstrate the ability of these cells to integrate into the ovarian environment, support follicle development, and restore normal ovarian function. To further improve the efficiency and fidelity of TICs’ generation from MSCs, it is important to optimize differentiation protocols by identifying novel growth factors and signaling pathways involved in TIC differentiation.

When exploring potential underlying mechanisms, it is advantageous to examine markers associated with fibroblast activation. Given the involvement of fibroblast activation in cell differentiation and the significant resemblance between fibroblasts and MSCs [49,50], investigating these markers could provide valuable insights. Future studies could incorporate specific fibroblast activation markers, such as α-SMA and FSP1, to better understand the differentiation pathways and the interplay between fibroblasts and MSCs. This approach could help clarify the role of fibroblast activation in the differentiation process and potentially improve the efficiency and specificity of TIC differentiation from MSCs.

Furthermore, exploring 3D culture systems and co-culturing with other ovarian cell types may enhance the differentiation process. These advanced culture techniques can more accurately mimic the in vivo ovarian microenvironment, thereby improving the functionality and therapeutic potential of differentiated TICs.

The self-renewal ability of MSCs, along with their convenient isolation from various tissues, such as bone marrow, adipose tissue, and peripheral blood, position them as an attractive source for TIC differentiation [51]. This paves the way for a comprehensive exploration of TICs, leading to a broader knowledge of these cells. 

## Figures and Tables

**Table 1:** Composition of in vitro TIC differentiation medium.

Reagent	Concentration	Supplier
Dulbecco’s modified Eagle’s medium/Ham’s nutrient mixture F-12 + GlutaMAX (DMEM/F12)	N/A	Thermo Fisher Scientific, Merelbeke, Belgium
KnockOut Gibco^TM^ serum replacement (KSR)	10% (*v*/*v*)	
Antibiotic–antimycotic (Anti–Anti)	1% (*v*/*v*)	
Insulin-transferrin-selenium (ITS)	1% (*v*/*v*)	
Recombinant human stem cell factor (SCF)	100 ng/mL	
Recombinant human bone morphogenic protein 6 (BMP-6)	20 ng/mL	
Recombinant human transforming growth factor beta (TGF-β1)	20 ng/mL	
Recombinant human hepatocyte growth factor (HGF)	20 ng/mL	
Recombinant human keratinocyte growth factor (KGF)	20 ng/mL	
Recombinant human insulin-like growth factor 1 (IGF-1)	100 ng/mL	
Recombinant Human fibroblast growth factor basic (bFGF)	10 ng/mL	
Recombinant human epidermal growth factor (EGF)	20 ng/mL	
Recombinant human growth differentiation factor 9 (GDF-9)	20 ng/mL	Sigma-Aldrich
Follicle-stimulating hormone (FSH)	100 ng/mL	Menopur, Ferring, Aalst, Belgium
Luteinizing hormone (LH)	100 ng/mL	

**Table 2:** List of used RT-qPCR probes.

Gene Name	Gene Symbol	Reference	Supplier
Steroidogenic acute regulatory protein	StAR	Hs00986559_g1	Thermo Fisher Scientific
Alanyl aminopeptidase	ANPEP (CD13)	Hs00174265_m1	
Perilipin 2	PLIN2	Hs00605340_m1	
Dipeptidyl peptidase 4	DDP4 (CD26)	Hs00897386_m1	
Peptidylprolyl isomerase A	PPIA	Hs01565699_g1	
Glyceraldehyde-3-phosphate dehydrogenase	GAPDH	Hs02758991_g1	
5′-nucleotidase ecto	NT5E (CD73)	Hs00159686_m1	
Thy-1 cell surface antigen	THY1 (CD90)	Hs06633377_s1	
Endoglin	ENG (CD105)	Hs00923996_m1	
CD44 molecule	CD44	Hs01075864_m1	
Platelet-derived growth factor receptor alpha	PDGFRA	Hs00998018_m1	
Nerve growth factor receptor	NGFR	Hs00609976_m1	

**Table 3:** List of used flow cytometry markers.

CD Marker	Target Cell	Supplier
CD73	MSC	Biolegend, Amsterdam, The Netherlands
CD90	MSC	
CD105	MSC	
CD34	MSC	
CD45	MSC	
HLA-DR	MSC	
CD13	TIC	

**Table 4:** List of used markers for immunocytochemistry.

Protein Name	Protein Symbol	Target Cell	Supplier
Cytochrome P450 family 17A1	CYP17A1	TICs	Biorbyt Ltd., Cambridge, UK
Insulin-like growth factor 1	IGF1		Biolegend, Amsterdam, The Netherlands
Platelet-derived growth factor receptor alpha	PDGFRA	MSCs	Abcam, Cambridge, UK
Nerve growth factor receptor	NGFR		

## Data Availability

All data generated or analyzed during this study are included in this published article.
